# 

**Published:** 1857-01

**Authors:** 


					THE
AMERICAN JOURNAL
DENTAL SCIENCE.
EDITED BY
CHAPIN A. HARRIS, M. D., D. D. S.
Ac' SNOWDEN PIGGOT, M. D.
VOL. *7?UNT E W SERIES.
PHILADELPHIA:
LINDSAY & BLAKISTON.
LONDON:
TRUBNER & CO. 12 PATERNOSTER ROW.
1857.
w
1
A
fA
k.
5

				

